# Research Progress on High-Temperature-Resistant Electromagnetic Wave Absorbers Based on Ceramic Materials: A Review

**DOI:** 10.3390/nano15040268

**Published:** 2025-02-11

**Authors:** Kangkang Tang, Feihang Long, Fenghua Zhang, Hongyuan Yin, Jiuzhou Zhao, Maoqian Xie, Ying An, Weimin Yang, Baihong Chi

**Affiliations:** 1 School of Mechanical and Electrical Engineering, East Campus, Beijing University of Chemical Technology, Beijing 100029, China; 2 Advanced Materials and Energy Research Center, China Academy of Aerospace Scienceand Innovation, Beiiing 100063, China

**Keywords:** ceramics, electromagnetic wave absorbing, metamaterial, 3D printing, high temperature resistance

## Abstract

Ceramic materials have the merits of an adjustable dielectric constant, high strength, high temperature resistance, and oxidation resistance, and are thus being used as the protection matrix for carbon series, metal oxides, and other wave-absorbing materials at high temperatures. Here, progress on high-temperature-resistant wave-absorbing ceramic materials is introduced through the aspects of their composition and structure. In addition, metamaterials used for such purposes, which are mainly produced through 3D printing, are also highlighted. The pros and cons of high-temperature-resistant electromagnetic wave absorbers based on ceramic materials are systematically analyzed, and possible development directions are proposed. This work may assist in the design and manufacture of a new generation of radars, ships, and aircraft.

## 1. Introduction

In recent years, the tug-of-war between radar detection and counter-detection has become one of the focuses of modern high-tech warfare. With the continuous improvement of the cooperative strike ability of systems for radar detection or missile interception, the stealth performance and high-speed maneuvering performance of airborne weapons and equipment are facing rigorous challenges. As an aircraft accelerates, its surface temperature increases due to friction with the air. In addition, the exhaust nozzles and nose cones of fighter jets can even reach temperatures as high as the thousands of degrees Celsius. They strongly reflect radar waves at high temperature, inducing a major threat [1]. At present, the most widely used technology in the field of radar stealth is coating or installing absorbant materials on the surfaces of airborne weapons and equipment to reduce radar echoes. Therefore, high-temperature-resistant materials with excellent microwave-absorbing capabilities have apparent practical significance.

Ceramic-based absorbing materials have become a research hotspot due to their high temperature resistance and structural stability [2]. In stealth fighter jets, ceramic absorbent coatings are applied to engine nozzles and high-temperature areas to reduce radar scattering characteristics [2,3,4]. During atmospheric re-entry, spacecraft face high temperatures and strong electromagnetic interference, so ceramic absorbing materials can simultaneously address the needs of high-temperature insulation and electromagnetic shielding. To ensure the electromagnetic compatibility of high-precision communication satellites in high-radiation environments, ceramic-based absorbent liners are typically installed inside satellite equipment to absorb reflected electromagnetic waves and prevent interference with the signals of internal devices [5].

Ceramic materials have a changeable dielectric constant [6,7,8], high temperature resistance [9,10,11], excellent chemical durability and oxidation resistance [12,13,14], and are suitable for microwave absorption at high temperatures. An ideal microwave absorber needs a low reflection coefficient, a small thickness, and to be effective in terms of the absorption of broadband electromagnetic waves [15,16,17,18,19]. However, the design and manufacture of such ideal microwave absorbers remain a huge challenge. Much research has been devoted to adjusting the composition of different ceramics and their hybridization with other materials, tuning their micro-/nano-structure, and altering metamaterials for the achievement of high performance in electromagnetic wave (EMW) absorption [20].

Wave-absorbing metamaterials regulate their equivalent electromagnetic parameters via the design of the structure of the material cell [21], thus achieving good impedance matching with free space, opening up new opportunities for EMW-absorbing materials to achieve significant absorption properties at specific or wide frequencies [22,23,24,25,26]. Wave-absorbing metamaterials overcome the bottleneck of traditional wave-absorbing materials in low frequency bands. They also possess strong absorption, small thickness, a flexible design, light weight, etc., all of which mean they have great potential application value in stealth technology. Traditional ceramic processing methods like powder sintering make it difficult to meet the design requirements of flexible and complex structural units of absorbent metamaterial structures; their manufacturing process is complex, and their cost is high [27,28]. As a highly flexible manufacturing technology [29], 3D printing technology has the strengths of a high material utilization rate, a rich material system, easy-to-form complex parts, and low cost, and it can achieve the fast processing of three-dimensional complex metamaterial structures. The integrated manufacturing of functional structures made of high-temperature-absorbing ceramic metamaterials with complex electromagnetic control functions has been explored for use in the future [30,31].

**Figure 1:**
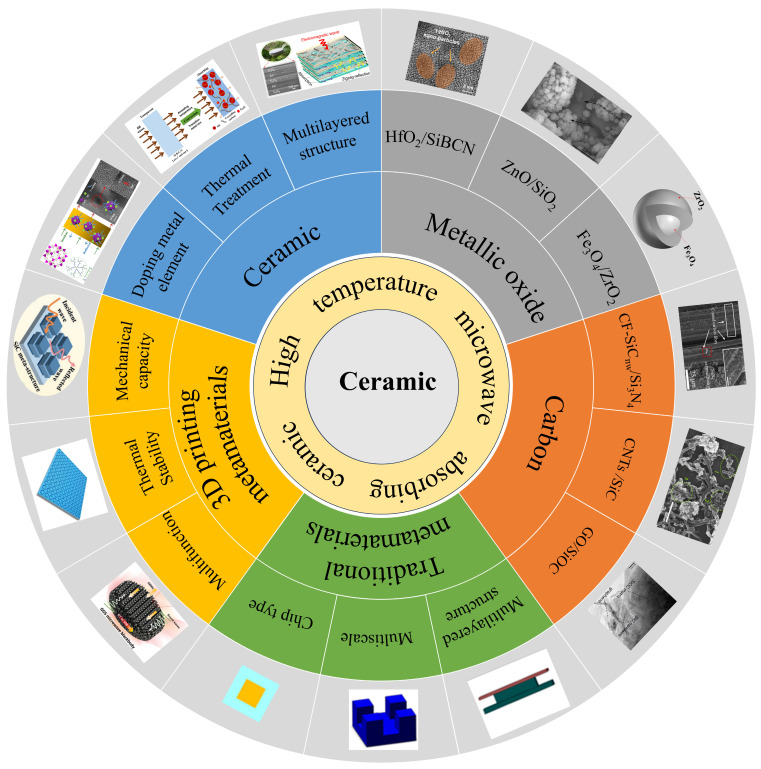
High-temperature-resistant ceramic materials for absorbing microwaves [32,33,34,35,36,37,38,39,40,41,42,43]. Reproduced with permission from Ref. [32]. Copyright 2016, Elsevier. Reproduced with permission from Ref. [33]. Copyright 2015, American Chemical Society. Reproduced with permission from Ref. [34]. Copyright 2020, Elsevier. Reproduced with permission from Ref. [36]. Copyright 2013, The American Ceramic Society. Reproduced with permission from Ref. [37]. Copyright 2023, American Chemical Society. Reproduced with permission from Ref. [38]. Copyright 2021, American Chemical Society. Reproduced with permission from Ref. [39]. Copyright 2015, Wiley. Reproduced with permission from Ref. [40]. Copyright 2020, Elsevier. Reproduced with permission from Ref. [41]. Copyright 2022, Wiley. Reproduced with permission from Ref. [42]. Copyright 2022, Springer Nature. Reproduced with permission from Ref. [43]. Copyright 2022, Wiley.

The current research progress on ceramic-based high-temperature-resistant absorbing materials (Figure 1) is summarized here, which mainly includes the following five parts: (1) concept and design; (2) typical studies on composites of ceramics with carbon or metal oxides or other ceramics; (3) metamaterials; (4) 3D-printed ceramics; (5) a summary and the prospects of the field.

## 2. Design of Microwave-Absorbing Materials

### 2.1. Absorbing Theory

**Figure 2:**
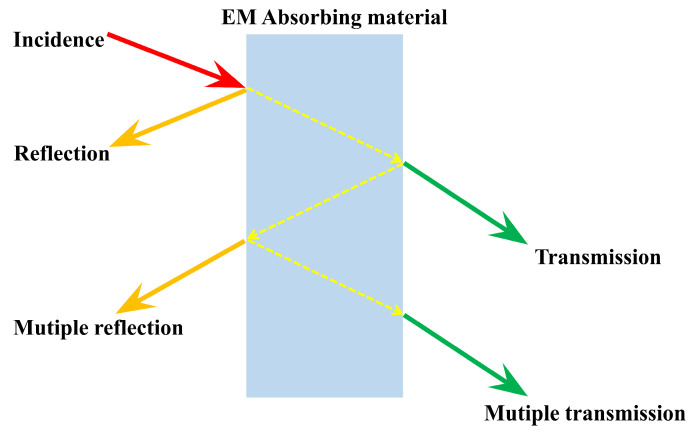
Interaction of an incident wave with a material.

EMW-absorbing materials can convert incident EMWs into heat through electromagnetic loss, with extremely low reflectivity and transmittance. The transmission process of the incident EMW in the medium is shown in schematic Figure 2. The ratios of the reflected, the absorbed, and the transmitted to the incident EMW are defined as reflectance (*R*), absorbance (*A*), and transmittance (*T*), respectively. The absorbance is expressed as follows [44]:

(1) A=1−R−T

Based on the theory of transmission lines [45], the reflection coefficient of EMW on the material Γ  can be expressed as follows:

(2) $$\Gamma =\frac{Z_{\mathrm{in}\;}-Z_{0}}{Z_{\mathrm{in}\;}+Z_{0}}$$

where *Z*_0_ and *Z_in_* represent the impedance in free space and that of the material, respectively. When the absorbing material is loaded with a metal backplane, according to the theory of transmission lines [45], *Z_in_* can be calculated as follows:

(3) $$Z_{in}=Z_{0}\sqrt{\frac{\mu_{r}}{\varepsilon_{r}}}tanh\left(j\frac{2\pi fd\sqrt{\mu_{r}\varepsilon_{r}}}{c}\right)$$

where f  is the frequency of the incident EMW, d is the thickness of the absorbing material, and c  is the EMW speed in a vacuum. In addition, $\varepsilon_{r}$ is the relative dielectric constant, and $\mu_{r}$ is the relative permeability of the material, which can be expressed as follows [46]:

(4) $$\varepsilon_{r}=\varepsilon^{\prime }-j\varepsilon^{\prime \prime }$$

(5) $$\mu_{r}=\mu^{\prime }-j\mu^{\prime \prime }$$

where the real parts $\varepsilon^{\prime }\;$and $\mu^{\prime }$ represent the energy storage capacity of the material, while the imaginary parts $\varepsilon^{\prime \prime }\;$ and $\mu^{\prime \prime }\;$ represent its ability to consume the energy of EMWs. The reflection loss (*RL*) of an absorbing material can be calculated by the following formula [47,48,49]:

(6) $$RL=20log_{10}\left|\frac{Z_{in}-Z_{0}}{Z_{in}+Z_{0}}\right|$$

The ideal absorbing material should have excellent performance in impedance matching and wave attenuation. The impedance matching (*Z_in_* = *Z*_0_) results in the negligible reflection on the surface of the material ( Γ=0), and the EMWs can enter the material completely.

According to the quarter wavelength matching theory [50], material thickness can affect the EMW-absorbing performance:

(7) $$t_{m}=\frac{n\lambda }{4}=\frac{nc}{4f_{m}\sqrt{\left|\mu_{r}\varepsilon_{r}\right|}}\left(n=1,3,5\ldots \right)$$

where λ  represents the wavelength of EMW, $t_{m}$ denotes material thickness, and $f_{m}$ is the frequency associated with minimum *RL*.

### 2.2. EMW-Absorbing Design of Ceramic Materials at High Temperature

EMW-absorbing materials can be divided into three types: magnetic, conductive, and dielectric loss types [46]. Magnetic loss-type absorbing materials (ferrite) transform into paramagnets above the Curie temperature, thus losing their absorbing ability [51]. Conductive loss-type absorbing materials (carbon materials such as carbon black, carbon nanotubes, and graphite) are vulnerable to oxidation at high temperature, causing impedance mismatch and degradation of absorbing performance [52]. Dielectric loss-type absorbing materials (ceramic materials) have no Curie temperature limitation as compared to magnetic loss-type materials, and they have better resistance to high temperature and oxidation than conductive loss-type materials.

At high temperatures, ceramic materials demonstrate significant advantages over other dielectric loss-type materials such as polymer composites [53,54,55]. Ceramic materials possess excellent high-temperature stability, enabling them to operate reliably under high-temperature conditions. In contrast, polymer materials are susceptible to decomposition in such environments. In addition, polymer materials exhibit weaker mechanical properties, making them vulnerable to compression or impact damage. However, ceramic materials typically have superior mechanical strength, allowing them to maintain structural integrity even under extreme conditions. Therefore, regarding application at high temperature, ceramics are more suitable and additionally can be used as a protective substrate for other absorbing materials.

**Figure 3:**
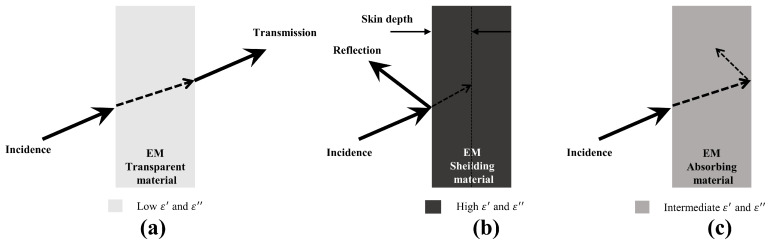
EMW transmission model of materials with different complex dielectric constants: (**a**) EM-transparent material model, (**b**) EM-shielding material model, (**c**) EM-transparent material model.

It has been shown that a combination of medium $\varepsilon^{\prime }$ and $\varepsilon^{\prime \prime }$ is beneficial to the absorbing performance of ceramic materials [1]. When they are relatively low ($\varepsilon^{\prime }=1∼5$,$\;\varepsilon^{\prime \prime }=0.01∼0.05$, Figure 3a), the material shows certain transmittance. When they are too high ($\varepsilon^{\prime }>20$, $\;\varepsilon^{\prime \prime }>10$, Figure 3b), the material presents electromagnetic shielding characteristics. Only when they are moderate ($\varepsilon^{\prime }=5∼20$,$\;\varepsilon^{\prime \prime }=1∼10$, Figure 3c) can the material achieve high performance in EMW absorption [56].

To enhance wave absorption efficiency at high temperatures, the design of ceramic-based composite materials typically integrates multiple loss mechanisms, including dielectric, conductive and magnetic loss. The dielectric constant and magnetic permeability of ceramic-based composite absorbing materials are both affected by temperature. According to Debye theory, the dielectric constant can be expressed as follows [57]:

(8) $$\varepsilon^{\prime }=\varepsilon_{\infty }+\frac{\varepsilon_{s-}\varepsilon_{\infty }}{1+\omega^{2}\tau {\left(T\right)}^{2}}$$

(9) $$\varepsilon^{\prime \prime }=\frac{\left(\varepsilon_{s-}\varepsilon_{\infty }\right)\omega \tau \left(T\right)}{1+\omega^{2}\tau {\left(T\right)}^{2}}+\frac{\sigma \left(T\right)}{\varepsilon_{0}\omega }$$

(10) $$\tau \left(T\right)=\tau_{0}e^{U/KT}$$

where $\varepsilon_{\infty }$ is the limited high-frequency permittivity, $\varepsilon_{s}$ is the static permittivity, $\varepsilon_{0}$ is the permittivity of free space, ω is the angular frequency, $\tau \left(T\right)$ is the temperature-dependent relaxation time,$\;\sigma \left(T\right)$ is the temperature-dependent electrical conductivity, $\tau_{0}$ is the prefactor, U is the activation energy, K is the Boltzmann constant, and T is the temperature.

In the low-temperature range, dielectric loss mainly originates from the polarization mechanism. As temperature rises, ion motion and interfacial polarization intensify, thus increasing the loss [58]. In the mid-temperature range, the polarization relaxation time gradually shortens, potentially causing a peak in dielectric loss at a certain frequency [59]. In the high-temperature range, conductive loss may become the dominant factor. Dielectric loss increases significantly with rising temperature, while polarization related dielectric loss may decrease [60].

Magnetic loss is related to saturation magnetization ($M_{s}$). Within a certain temperature range, $M_{s}$ remains relatively stable. However, as the temperature continues to rise, it decreases sharply. When the temperature reaches the Curie temperature, $M_{s}$ approaches zero [61]. At this point, according to Equation (11), the magnetic permeability also tends toward zero, and the magnetic loss almost disappears [62].

(11) $$\mu \left(T\right)=\frac{4}{3}\pi r^{3}M_{s}\left(T\right)$$

where $\mu \left(T\right)$ is the temperature-dependent permeability, r is the radius of the magnetic nanoparticle, $\mathrm{and}\;M_{s}\left(T\right)$ is the temperature-dependent saturation magnetization.

## 3. Composite Materials of Ceramic Matrix with Different Agents

The EMW-absorbing performance can be improved by optimizing the material composition as the absorbing ability of a single material is limited. Ceramic-based high-temperature wave-absorbing materials are made of a low dielectric wave-transmitting ceramic matrix and a wave-absorbing agent. Common matrix ceramics include Si_3_N_4_, SiO_2_, SiCN, SiBCN, etc. Common absorbing agents include carbon materials (like carbon nanotubes, graphene, and carbon fiber), metal oxides (like ZnO and Fe_3_O_4_), ceramic materials (like SiC, SiOC, and SiBCN), and their composites, which are elucidated in the following sections.

### 3.1. Composites of Ceramics and Carbon-Based Materials

Carbon-based materials have great potential in EMW absorption. As lightweight, low-cost and highly conductive materials, their rich and unique structures provide a variety of options for preparation processes. However, carbon materials are easily oxidized at high temperatures; thus, they cannot meet the stringent requirements in the field of wave absorption. Pure carbon has unsatisfactory wave absorption performance, with a narrow frequency band, a high reflection loss value, and no adaptability to high-temperature environments, etc. Therefore, it is usually distributed in high-temperature-resistant ceramic matrices to obtain high-temperature wave absorption composite materials (as shown in Table 1).

**Figure 4:**
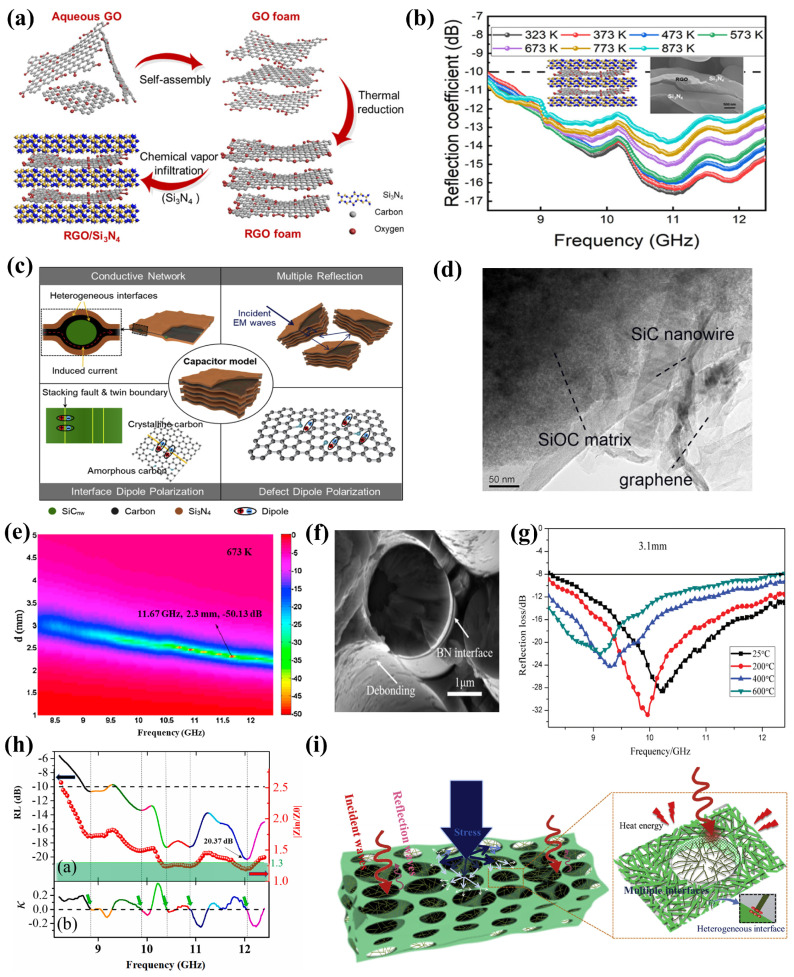
(**a**) Schematic diagram of the preparation route of RGO/Si_3_N_4_. Reproduced with permission from Ref. [63]. Copyright 2019, American Chemical Society. (**b**) *RC* curves of RGO/Si_3_N_4_ with a thickness of 4.3 mm at different temperatures. Reproduced with permission from Ref. [63]. Copyright 2019, American Chemical Society. (**c**) Schematic diagram of EMW absorption principle of SiCnw/C/Si_3_N_4_. Reproduced with permission from Ref. [65]. Copyright 2019, Elsevier. (**d**) TEM image of GO/SiOC ceramics containing 3 wt% GO. Reproduced with permission from Ref. [32]. Copyright 2016, Elsevier. (**e**) Reflection coefficient of 2 wt% GO/SiOC at a temperature of 673 K and with a thickness of 2.3 mm. Reproduced with permission from Ref. [32]. Copyright 2016, Elsevier. (**f**) Surface morphology of BN-coated SiC_f_/AlPO_4_ at 1273 K. Reproduced with permission from Ref. [66]. Copyright Taylor & Francis. (**g**) Reflection loss curves with a thickness of 3.1 mm SiC_f_/BN/AlPO_4_/MWCNTs at different temperatures. Reproduced with permission from ref. [66]. Copyright Taylor & Francis. (**h**) The upper part (a) is the selected optimal reflection loss of CF-SiC/Si_3_N_4_ (colored line) and corresponding normalized input impedance *|Z_in_/Z*_0_*|* (red scatter plot) at 1073 K. The lower part (b) is the calculated relaxation factor *k* of CF-SiC/Si_3_N_4_ based on experimental data at 1073 K. Reproduced with permission from Ref. [67]. Copyright 2020, Elsevier. (**i**) Schematic diagram of mechanism of EMW attenuation and enhancement of mechanical properties. Reproduced with permission from Ref. [34]. Copyright 2020, Elsevier.

Graphene has light weight, moderate electrical conductivity, a unique two-dimensional structure and excellent mechanical strength, which is needed in the preparation of EMW-absorbing materials [69,70,71]. Due to its abundant defects and functional groups, the conductivity of reduced graphene oxide (RGO) is much lower than that of graphene, so the preparation of EMW-absorbing materials using RGO is more favored by researchers [72,73,74]. Hou et al. [63] prepared a composite material of RGO and silicon nitride (Si_3_N_4_) ceramics by combining an improved freeze-drying method with chemical vapor infiltration (CVI) (Figure 4a). Si_3_N_4_ is penetrated by CVI into the porous RGO, and in situ growth occurs in the surface of RGO sheets, ensuring the stability of RGO at high temperatures. The sandwich structure of the composite material provides rich interfaces and increases the interfacial polarization of the material, which is conducive to EMW attenuation. When the amount of absorbant content in RGO is as low as 0.16 wt%, the effective absorption bandwidth (EAB, *RL* < −10 dB) can cover the entire X-band (8.2~12.4 GHz) in the temperature range of 323~873 K, and the electromagnetic absorption performance is less affected by temperature (Figure 4b) [63].

Compared with traditional graphene/ceramic composites, the integration of graphene oxide in polymer-derived ceramics forms a new strategy for the preparation of high-performance electromagnetic absorbent materials at high temperatures. Han et al. [32] prepared high-performance EMW absorption composites using graphene and polysiloxane-derived SiOC ceramics. The addition of appropriate amount of graphene promoted nucleation of SiC nanowires and formed a layered structure of two-dimensional (2D) graphene and one-dimensional (1D) SiC nanowires in the SiOC ceramic matrix. Defects and residual functional groups in graphene oxide, layer faults and connection points in SiC nanowires, and conductive networks (Figure 4d) enabled efficient EMW absorption at high temperatures in ceramic matrix composites. With a thickness of 2.3 mm, the composite materials displayed a *RL_min_* of −50.13 dB at a temperature of 673 K and an effective absorption bandwidth of 3.9 GHz in the X-band (Figure 4e) [32].

Carbon nanotubes play an important role in the field of wave-absorbing materials because of their excellent properties such as high electrical conductivity, high mechanical strength and thermal stability, and light weight [75,76,77,78,79]. By modifying carbon nanotubes with dielectric materials, the dielectric constant can be well adjusted to improve the microwave attenuation characteristics. Wan et al. [66] applied multi-wall carbon nanotubes (MWCNTs) as absorbers to mix BN-coated SiC fibers with AlPO_4_ slurry containing MWCNTs and formed SiC_f_/BN/AlPO_4_/MWCNTs composites by hot-pressing (Figure 4f). In the temperature range of 298~873 K, when the sample thickness was 3.1 mm, the *RL_min_* was −22 dB, and the effective absorption bandwidth in the X-band was about 3.7 GHz (Figure 4g) [66]. In addition, due to the introduction of BN phase, the flexural strength and fracture displacement of composite materials were significantly improved, and the brittle fracture behavior was transformed into ductile fracture behavior. This provides valuable information for exploring and developing advanced microwave-absorbing materials in industry.

The introduction of enhanced interfacial polarization is an effective way of adjusting the dielectric properties of materials with temperature. Lan et al. [34] constructed a silicon carbide nanowire network on a porous silicon carbide skeleton by carbothermal reduction and successfully prepared carbon nanotube-derived porous silicon carbide with multiple interfaces. Due to the synergistic structural effect of the porous structure and the nanowires, the obtained binary SiC sample exhibited remarkable EMW-absorbing properties at high temperatures (Figure 4i) [34]. Meanwhile, the construction of SiC nanowire network greatly improved the compressive strength of the material. Therefore, new synthetic carbon nanotube-derived binary silicon carbide has great application prospects in terms of actual electromagnetic absorption in harsh environments.

Carbon fiber has advantages in its electrical conductivity and thermal and mechanical properties. Structural design is effective in realizing temperature insensitivity, which is conducive to the practical application of EMW absorption at high temperatures [80,81,82]. Liang et al. [67] prepared CF-SiCnw/Si_3_N_4_ composites with a layered structure by using catalytic chemical vapor deposition (CVD) and cold-pressing sintering and introduced the relaxation factor *k* for the first time to predict temperature-dependent multiple relaxation behavior (Figure 4h) [67].

Xiao et al. [65] prepared SiCnw/C/Si_3_N_4_ layered porous ceramic composites with excellent EMW absorption properties by freeze-drying, carbonization, and CVI (Figure 4c). It has the characteristics of high absorption rate (more than 39.7 dB), wide absorption band (covering the whole X-band), low density (0.92 g/cm^3^), small thickness (2.9 mm), and high-temperature stability (298~873 K). Its unique structure was not only conducive to EMW absorption but also obtained a compressive strength of up to 168 MPa [65]. The special multistage porous structure creates a long and complex channel for the incoming EMW, so that it is repeatedly scattered inside, thus improving the material’s electromagnetic wave absorption capacity.

### 3.2. Composites of Ceramics and Metal Oxides

Metal oxide materials possess outstanding magnetic loss characteristics. However, when its temperature is above Curie temperature, it will become a paramagnet and lose its wave absorption performance, so it is generally hybridized with ceramic materials to optimize its high-temperature wave absorption performance, as summarized in Table 2.

**Figure 5:**
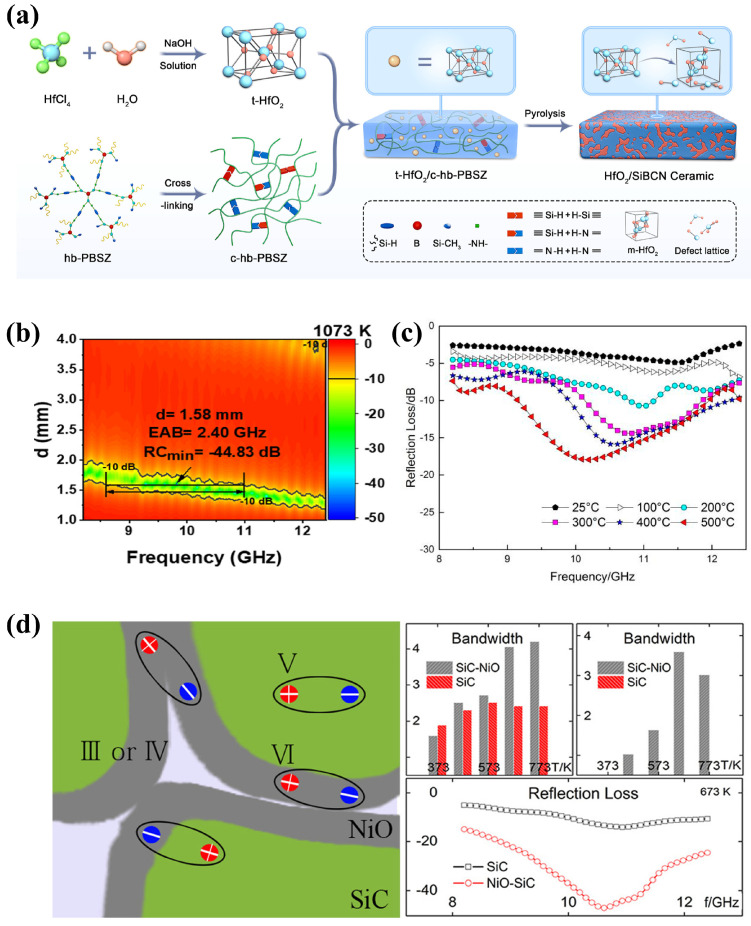
(**a**) HfO_2_/SiBCN ceramics prepared by hydrothermal synthesis of t-HfO_2_ nanoparticles and co-pyrolysis of t-HfO_2_/c-hb-PBSZ complexes. Reproduced with permission from Ref. [37]. Copyright 2023, American Chemical Society. (**b**) Reflection loss of HfO_2_/SiBCN ceramics at 1073 K temperature and 1.58 mm thickness. Reproduced with permission from Ref. [37]. Copyright 2023, American Chemical Society. (**c**) Reflection loss of Al_2_O_3_-MoSi_2_-Cu composite coating at 773 K temperature and 1.7 mm thickness. Reproduced with permission from Ref. [83]. Copyright 2014, Springer Nature. (**d**) NiO-SiC multipolarization (left), absorption bandwidth of SiC and NiO-SiC at different temperatures (right). Reproduced with permission from Ref. [84]. Copyright 2015, American Chemical Society.

The addition of dopants to metal oxide nanoparticles can enhance both the electromagnetic wave absorption capabilities and the mechanical strength of ceramic composites. Yan et al. [37] prepared HfO_2_/SiBCN ceramics by co-pyrolysis crosslinking and high-branched poly-borosilazane precursors and HfO_2_ nanoparticles (Figure 5a). Introducing HfO_2_ nanoparticles decreases the porosity of ceramics, thereby increasing their density and enhancing their mechanical properties. In addition, the synergistic effect of phase transition and lattice defects caused by the interaction of HfO_2_ nanoparticles with SiBCN ceramic particles improved the interface and defect polarization, and the randomly distributed, finger-like carbon also facilitated the creation of conductive networks, resulting in conductivity losses and improved EMW absorption performance. At a high temperature of 1073 K and a sample thickness of 1.58 mm, *RL_min_* reached −44.83 dB, and the effective absorption bandwidth was 2.4 GHz within the X-band (Figure 5b). HfO_2_ nanoparticles simultaneously improved the mechanical strength and electromagnetic absorption performance of SiBCN ceramics. This offered valuable insights for the production of brittle ceramics that exhibit high ductility and superior electromagnetic properties. [37].

Transition metal oxides (such as Fe_3_O_4_, NiO, Cu_2_O, etc.) are often used as fillers for high-temperature absorbing ceramic composites because of their high dielectric and magnetic loss, and the presence of transition metal elements can catalyze the formation of nanocrystals and regulate the dielectric performance of ceramic materials [20,83,84]. Gao et al. [83] fabricated an Al_2_O_3_-MoSi_2_-Cu composite coating using a plasma-spraying technique. In the high-temperature environment, the copper in the coating was oxidized to Cu_2_O, thus enhancing the wave absorption capability of the composite at high temperatures. When the temperature was 773 K and the sample thickness was 1.7 mm, the *RL_min_* was −17.96 dB and the effective absorption bandwidth was 2.42 GHz (Figure 5c) [83]. Yang et al. [84]. successfully prepared NiO nanolayers on SiC with a two-step method, thus significantly improving the dielectric properties of SiC, and the prepared NiO-SiC composite had excellent microwave absorption performance under the synergistic effect of multiple relaxation and conductance (Figure 5d), providing a novel and effective way of regulating microwave absorption.

Core–shell nanomaterials can take into account multiple loss mechanisms like dielectric loss, magnetic loss, and resistance loss [86]. The interface and hierarchical structure of the material were increased, and the scattering loss and absorption performance of the material were enhanced by introducing a core–shell structure. Yu et al. successfully synthesized Fe_3_O_4_@ZrO_2_ with a yolk–shell structure with a uniform zirconia shell thickness through the sol–gel method, assisted by the polymer surfactant hydroxypropyl cellulose. This material had the stability of a ceramic material due to the ZrO_2_ shell and had the ferromagnetic loss ability due to Fe_3_O_4_. It not only maintained structural stability in 298~1273 K but also showed high-temperature absorption stability from room temperature to 773 K [35].

The incorporation of metal oxide nanoparticles into polymer-derived ceramics offers a new way of preparing high-performing electromagnetic absorbent materials at high temperatures. Luo et al. [20] designed and tuned graphene@Fe_3_O_4_/SiBCN nanocomposites with a unique A/B/C structure through the polymer-derived ceramics (PDC) pathway, using graphene@Fe_3_O_4_ and liquid polyborosilane (PBSZ) as raw materials. Transition metal atoms in magnetic Fe_3_O_4_ nanoparticles induced the in situ formation of nanocrystals, including metal silicides, vortex-layer carbon, or silicon carbide (SiC), thereby regulating the complex dielectric constant and dielectric loss. In addition, the ternary synergistic effect of A, B, and C phases enhanced the oxidation resistance at high temperature and the EMW absorption of the material. SiBCN (phase A) acted as a substrate to prevent graphene@Fe_3_O_4_ from being oxidized. Even at a high temperature of 873 K, the nanocomposite also exhibited effective EMW absorption, with an *RL_min_* of −46 dB and an EAB covering 93.6% in the X-band. In addition, since the liquid PBSZ preceramic precursor contained UV-curable vinyl, the nanocomplex combined with 3D printing technology formed thermal parts with complex structures, thus showing great application potential [20].

### 3.3. Ceramic Composites

Ceramics like SiC, SiCN, SiOC, and SiBCN have the advantages of low relative density, an adjustable dielectric constant, high mechanical strength, high thermal stability, and resistance to oxidation. They can be used not only as the substrate of high-temperature absorbant ceramic composite materials, but also as absorbing agents. However, the wave absorption performance of pure ceramic is usually not good enough at high temperatures. Therefore, doping with transition metal elements (Fe, Co, Ni, etc.), fabricating into a multilayer structure, surface modification, and high-temperature heat treatment (Table 3) are usually used to improve its dielectric constant and broaden the effective absorption band for regulating its wave absorption performance to meet the needs of applications.

**Figure 6:**
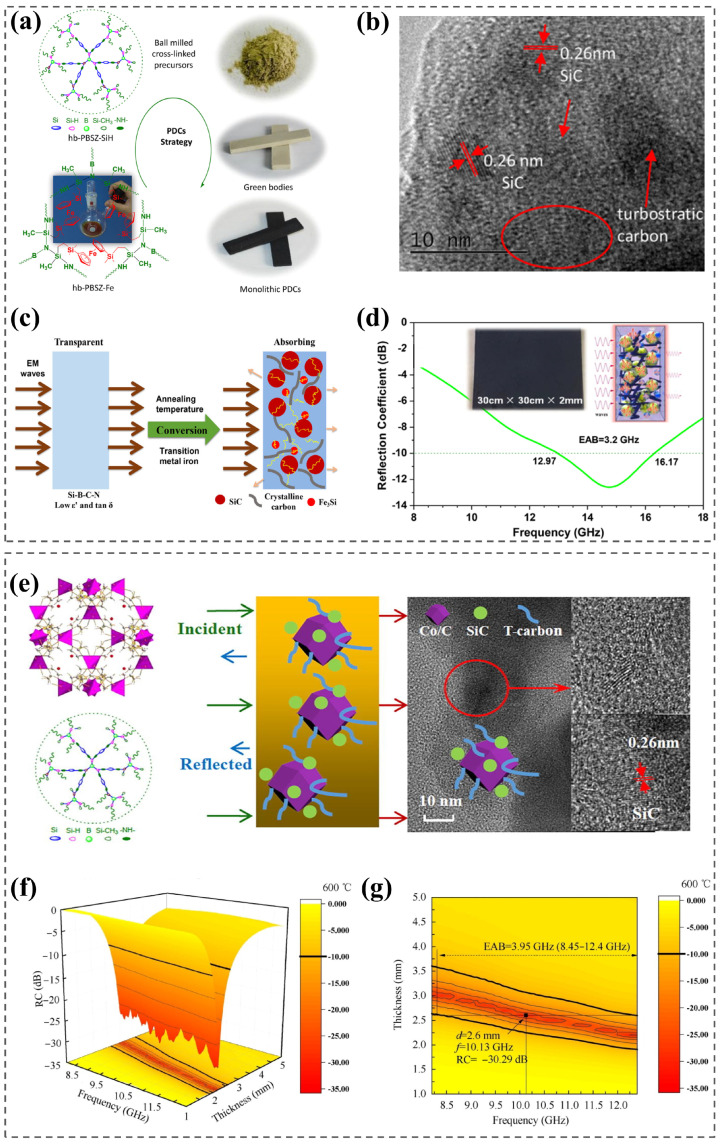
(**a**) Preparation of iron-containing SiBCN monolithic ceramics using a polymer-derived ceramic (PDC) strategy. Reproduced with permission from Ref. [39]. Copyright 2018, Wiley. (**b**) TEM images of iron-containing SiBCN ceramics. Reproduced with permission from Ref. [39]. Copyright 2018, Wiley. (**c**) Schematic of the EMW absorption mechanism of SiBCN monolithic ceramics by annealing and transition metal assistance. Reproduced with permission from Ref. [39]. Copyright 2018, Wiley. Copyright 2018, Wiley. (**d**) Reflection loss curve of ferro SiBCN ceramics at 1158 K temperature. Reproduced with permission from Ref. [39]. Copyright 2018, Wiley. (**e**) Preparation of ZIF67/SiBCN nanocomposites with high temperature EMW absorption by polymer-derived ceramics strategy. Reproduced with permission from Ref. [40]. Copyright 2021, Elsevier. (**f**) Three-dimensional images of theoretical *RC* values calculated at 873 K temperature. Reproduced with permission from Ref. [40]. Copyright 2021, Elsevier. (**g**) Two-dimensional projection image of theoretical RC values calculated at 873 K temperature. Reproduced with permission from Ref. [40]. Copyright 2021, Elsevier.

A nanocrystalline phase can be formed in situ by introducing a transition metal into the ceramic matrix and increasing the annealing temperature in order to adjust the complex dielectric constant and dielectric loss tangent. Therefore, transition metal-modulated ceramic materials can achieve impedance matching and EMW attenuation even at high temperatures. Luo et al. [39] prepared SiBCN monolithic ceramics containing iron using soluble hyperbranched polyborosilazane as a preceramic precursor (Figure 6a). After the addition of iron to the SiBCN ceramic matrix and an increase in the annealing temperature, nano-crystalline phases like SiC and Fe_3_Si were generated in situ, which promoted the conversion of free carbon from a disordered state to graphitic carbon, further leading to the layered structure of the ceramics and producing a huge number of interfaces that contribute to the absorption of EMWs (Figure 6b,c). The ceramic absorbing material had an *RL_min_* of −12.6 dB and an EAB of 3.2 GHz in a high-temperature environment of 1158 K (Figure 6d), with high temperature resistance as well as excellent mechanical properties [39].

They also prepared a cobalt-containing silicon-boron-carbonitriding nanomaterial (MOF/SiBCN) by pyrolysis of a metal–organic skeleton (ZIF-67) and hyperbranched polyborosilazane. The rhombohedral dodecahedron ZIF-67 and cobalt elements promoted the formation of dielectric loss phases, enhancing the wave absorption (Figure 6e) [40]. In addition, ZIF-67/SiBCN nanomaterials exhibited excellent microwave absorption properties at both room temperature and high temperatures. At room temperature, the *RL_min_* was −51.6 dB, and the effective absorption bandwidth was 3.9 GHz. At a high temperature of 873K, the *RL_min_* was −30.3 dB, and the EAB was 4.0 GHz, nearly covering the entire X-band (Figure 6f,g) [40]. It can be seen that the ZIF-67/SiBCN nanocomposite is a promising microwave absorption material with high temperature resistance. It has great significance for the design of next-generation aero-engines and stealth aircraft.

**Figure 7:**
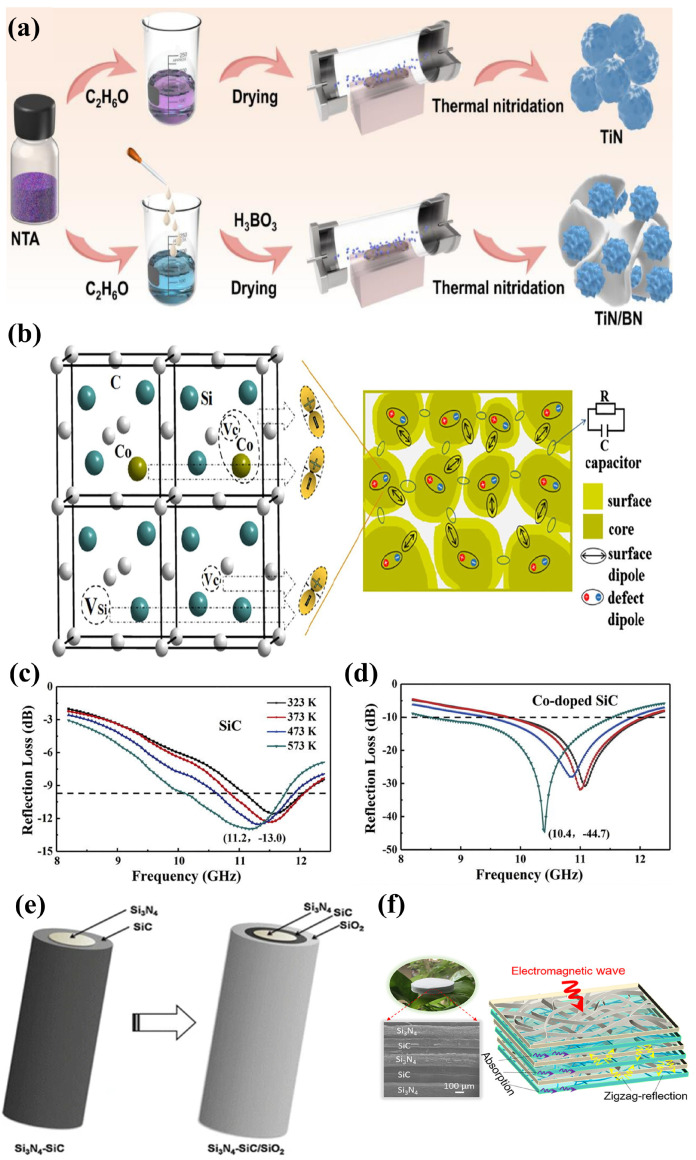
(**a**) Schematic diagram of synthesis of TiN and TiN/BN composites. Reproduced with permission from Ref. [87]. Copyright 2022, Elsevier. (**b**) Schematic diagram of possible polarization mechanisms leading to dielectric loss in the microwave band. Reproduced with permission from Ref. [92]. Copyright 2018, Elsevier: interfacial polarization of defective dipoles (left) and core–shell nanoparticles in Co-doped SiC (right); (**c**) Reflection loss curves of undoped SiC with a thickness of 1.7 mm at different temperatures. Reproduced with permission from Ref. [92]. Copyright 2018, Elsevier. (**d**) Reflection loss curves of Co -doped SiC with a thickness of 1.7 mm at different temperatures. Reproduced with permission from Ref. [92]. Copyright 2018, Elsevier. (**e**) Formation of Si_3_N_4_-SiC/SiO_2_. Reproduced with permission from Ref. [88]. Copyright 2018, Springer Nature. (**f**) Schematic diagram of wave absorption principle of alternating multilayer Si_3_N_4_/SiC ceramic aerogel. Reproduced with permission from Ref. [38]. Copyright 2021, American Chemical Society.

Kuang et al. [92] synthesized Co-doped SiC powders with heterogeneous core–shell nanostructures. Doped Co introduces an abundance of defects (carbon vacancies and silicon vacancies) in SiC that can act as dipoles to increase polarization losses and produce more carriers to enhance leakage losses (Figure 7b). In addition, core–shell nanostructures lead to interfacial polarization, which further increases dielectric loss and improves microwave absorption performance. Compared with undoped SiC, Co-doped SiC exhibited significantly increased high-temperature dielectric loss as well as microwave absorption properties in the 8.2–12.4 GHz range (Figure 7c,d) [92].

An ideal high-temperature EMW absorber should have excellent wave-absorbing performance and insensitivity to temperature change. Shi et al. [87] applied in situ synthesis to construct a composite of titanium nitride and boron nitride (TiN/BN), where TiN was the loss unit and BN was the impedance-matching unit (Figure 7a). BN not only effectively adjusted the conductivity and optimized the impedance matching but also provided the material with stable microwave absorption performance. Stable dielectric losses ensured that TiN/BN composites had stable high-temperature absorption properties in the X-band. Li et al. [88] prepared ceramic composite of Si_3_N_4_-SiC and SiO_2_ with a low–high–low dielectric constant layered structure (Figure 7e), where interlaced Si_3_N_4_ grains were covered by SiC nanocrystals, while SiC was covered by SiO_2_ films. As a result, there were a large number of interfaces between the grain boundaries between SiC-SiC, SiC-SiO_2_, and SiC-Si_3_N_4_, which enhanced the interfacial polarization and multiple reflections of EMWs, achieving an *RL_min_* of −35.9 dB and an EAB of 4.02 GHz at 873 K. Cai et al. [38] used a simple overlapping method to prepare high-performance EMW-absorbing ceramic aerogels composed of an alternating multilayer transparent Si_3_N_4_ layer and a wave-absorbing SiC layer. The multilayer alternating structure caused efficient “reflection–absorption–cyclic reflection” attenuation of EMWs (Figure 7f), which covered the entire X-band at temperatures as high as 473~1273 K and had an ultra-low density (~8 mg/cm^3^) [38].

**Figure 8:**
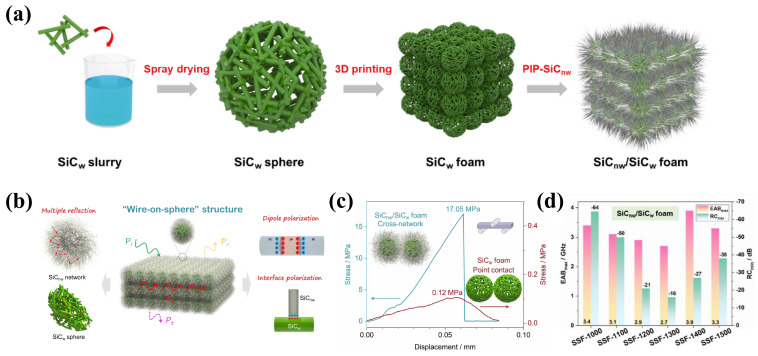
(**a**) Flow chart of the production process of SiCnw/SiCw foam. Reproduced with permission from Ref. [89]. Copyright 2022, Elsevier. (**b**) Schematic diagram of EMW absorption mechanism of the SiCw/SiC foam layered structure with a “wire-on-sphere” configuration. Reproduced with permission from Ref. [89]. Copyright 2022, Elsevier. (**c**) Stress displacement curves of SiCw foam and SiCnw/SiCw foam. Reproduced with permission from Ref. [89]. Copyright 2022, Elsevier. (**d**) Diagram of dielectric constant, maximum absorption bandwidth, and minimum reflection loss of SiCw/SiCw foam oxidized at 1273~1773 K for 1 h. Reproduced with permission from Ref. [89]. Copyright 2022, Elsevier.

Lv et al. [89] developed a catalyst-free precursor-infiltrated pyrolysis process to enable SiC nanowires (SiCnw) to grow on the SiC whisker (SiCw) spheres, thus achieving unique “wire-on-sphere” layered nanowires/whiskers of a SiC (SiCnw/SiCw) ceramic foam material (Figure 8a). Due to its unique structure, the SiCnw/SiCw foam had a density as low as 0.92 g/cm^3^, excellent bending strength of 17.05 MPa, and high-temperature wave absorption properties (Figure 8b,c). After oxidation at a 1273~1773 K temperature, the material still had an EAB of 2.7~3.9 GHz and an *RL_min_* of −16~−64 dB (Figure 8d) [89]. As a result, SiCnw/SiCw foam is a great candidate EMW-absorbing material for exhausts in cutting-edge aircrafts. The preparation process provides great inspiration for the design and manufacture of other EMW-absorbing micro-nano materials.

## 4. Ceramic-Based Wave-Absorbing Metamaterials

Metamaterials are a type of artificial array structure or material that have exceptional physical properties that natural materials do not have, and their special properties are due to the geometry and distribution of their unit structures. The appearance of metamaterials breaks the design principles of traditional materials. Metamaterials can overcome the performance limitations of existing materials only through the ordered arrangement of physical structures. Metamaterials can exhibit extraordinary physical properties such as refractive index, permeability and permittivity of negative value, and inverse Doppler effect, thus being widely used in stealth technology, electromagnetic interference, and other fields [93]. At present, the raw materials used to prepare wave-absorbing metamaterials are basically composed of metals and low-dielectric polymer materials, but the properties of metamaterials are mainly determined by their structural design rather than the properties of the raw materials themselves. Therefore, metamaterials can be prepared from various raw materials. Ceramic matrix composites, in particular, exhibit more natural advantages than metals, like resistance to high temperature, oxidation and corrosion, thus having great potential in wide range of applications [94].

### 4.1. Traditional Ceramic-Based Wave-Absorbing Metamaterials

The preparation of traditional ceramic absorbing metamaterials is mainly based on the molding of slurry and the formation of periodic unit metamaterials on a substrate by spraying patches. However, current research is mostly focused on their wave absorption performance at room temperature. To achieve high-temperature wave absorption, metamaterials can be made by using suitable conductive and dielectric materials with high temperature resistance. Table 4 summarizes the wave-absorbing properties of traditional ceramic-based wave-absorbing metamaterials at high temperatures.

Li et al. [95] designed a refractory metamaterial microwave absorber with conductive titanium diboride (TiB_2_) as the unit cell and dielectric alumina (Al_2_O_3_) as the interval cell. The metamaterial had strong microwave absorption properties over a wide range of temperatures, i.e., from room temperature to 1073 K. The impact of temperature on the dielectric constant of the Al_2_O_3_ spacer layer was small, in order to ensure almost constant absorption. This study provides a method of preparing materials with strong microwave-absorbing ability, showing the broad prospects of ceramic metamaterials in applications involving high temperatures. Gao et al. [96] prepared La_0.5_Sr_0.5_CoO_3_/Al_2_O_3_ ceramic sheets by a plasma-spraying process with periodic square and circular structures to enhance the microwave absorption performance. At a temperature of 773 K and in the X-band, the *RL* is below −10 dB [96].

Qiao et al. [100] studied a metamaterial absorber made of graphite microsheets and water glass. At a temperature of 673 K, the EAB covered the entire X-band. To meet even more demanding conditions, they firmly bonded a resistant frequency-selective surface array made of a blend of Al powder, MoSi_2_ powder, and water glass onto a quartz substrate by applying screen printing and sintering. The resulting metamaterial absorber displayed an EAB of 4 GHz (7~11 GHz) in a temperature range from room temperature to 873 K, showing great temperature insensitivity [101].

**Figure 9:**
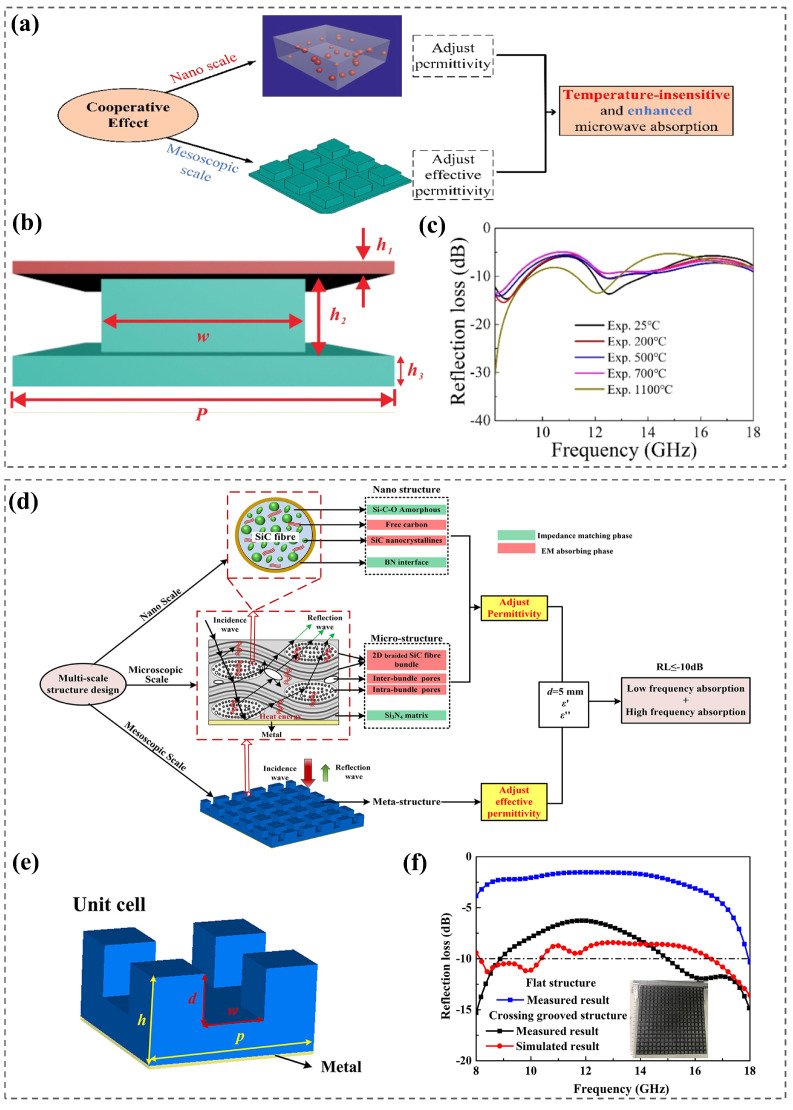
Schematic diagram of material design (**a**), three-dimensional diagram (**b**), and reflection loss temperature-insensitive curve (**c**) of TiB_2_/Al_2_O_3_/MgAl_2_O_4_ composite metamaterials. Reproduced with permission from Ref. [98]. Copyright 2022, Springer Nature. Multi-scale design scheme (**d**) and structure diagram (**e**) of SiC_f_/Si_3_N_4_ composite with cross-groove elements. Reproduced with permission from Ref. [99]. Copyright 2022, The American Ceramic Society. (**f**) Measured and simulated *RL* results. Reproduced with permission from Ref. [99]. Copyright 2022, The American Ceramic Society.

Yang et al. [98] prepared a metamaterial with layered SiO_2_ ceramics and patch-type and flat TiB_2_/Al_2_O_3_/MgAl_2_O_4_ composites, which had stable dielectric properties and achieved temperature-insensitive microwave absorption performance (Figure 9a,b). In the range of 298~1373 K and 8.2~18.0 GHz, the *RL* was below −5 dB, showing great potential for high-temperature radar stealth. Based on the multi-scale design combining a metamaterial with a microstructure (Figure 9d), Zhou et al. [99] prepared a SiC_f_/Si_3_N_4_ composite wave absorber with a cross-slot element structure (Figure 9e). Broadband absorption (8~18 GHz) with *RL* below −5 dB was achieved from room temperature to 773 K (Figure 9f).

### 4.2. 3D-Printed Ceramic Wave-Absorbing Metamaterials

The traditional processing method makes it difficult to manufacture the complex structure of metamaterials. Conversely, 3D printing has unique advantages in preparing complex structures. Recently, 3D printing has been used to prepare wave-absorbing metamaterials, mainly including fused deposition modeling (FDM) [102], selective laser sintering (SLS) [103,104,105], inkjet printing (IP) [106], direct ink writing (DIW) [107], stereolithography apparatus (SLA), and digital light processing (DLP) [108,109,110,111]. Thus, 3D printing ceramic-based wave-absorbing metamaterials has become a current research trend.

**Figure 10:**
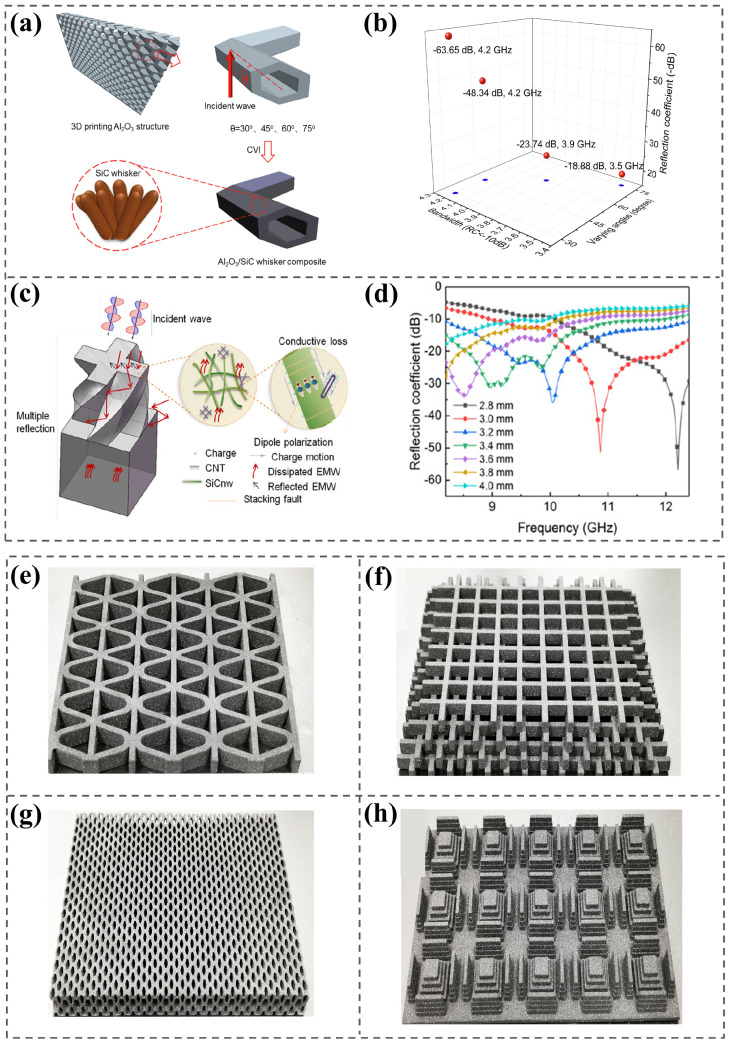
Schematic diagram (**a**) and comparison of the optimal-thickness EAB and *RC_min_* (**b**) of 3D-printed Al_2_O_3_/SiC whisker composites from different angles. Reproduced with permission from Ref. [112]. Copyright 2019, Elsevier. EMW absorption mechanism (**c**) and reflection loss curve (**d**) of Al_2_O_3_/CNT/SiCnw/SiOC composites with a twisted cross metamaterial structure. Reproduced with permission from Ref. [113]. Copyright 2019, Elsevier. Corrugated structure (**e**), gradient porous structure (**f**), skew honeycomb structure (**g**), and element structure (**h**) of 3D-printed SiCw/Si_3_N_4_ parts. Reproduced with permission from Ref. [114]. Copyright 2019, Elsevier.

Impedance matching and the attenuation principle are crucial to EMW-absorbing materials in achieving wider bandwidth and higher intensity. Through optimizing the macrostructure of metamaterials and adjusting the microstructure of the absorbing material, the impedance matching characteristics and absorption properties can be easily improved. Mie et al. [112] used SLA to prepare Al_2_O_3_/SiC whisker (SiCw) composite ceramic microwave-absorbing metamaterials with an inclined honeycomb array structure. The perforated inclined honeycomb structure designed at a reasonable angle and the micron-scale SiCw (Figure 10a) effectively improved impedance matching, internal scattering, and dielectric loss, thereby inducing excellent wave absorption performance. At an inclination of 30° and a sample thickness of 3.5 mm, the effective absorption bandwidth range covered the entire X-band with an *RL_min_* of −63.65 dB (Figure 10b), which provided a novel and efficient approach to fabricating a structural absorber with broader and higher absorbing performance [112]. They also prepared Al_2_O_3_/CNTs/SiCnw/SiOC with twisted cross-metamaterial structures using SLA and PIP [113]. CNTs enhanced the nucleation effect of SiCnw. By changing the content of carbon nanotubes, the microstructure of SiCnw was adjusted, and a conductive network structure was formed with SiCnw, which largely enhanced the dielectric loss and conductive loss (Figure 10c). When the thickness of the composite was 3.2 mm, the EAB covered the entire X-band, and the *RL_min_* was −35.95 dB (Figure 10d) [113]. Yu et al. [114] prepared 3D-printed SiCw/Si_3_N_4_ wave-absorbing ceramics with a corrugated structure (Figure 10e), gradient porous structure (Figure 10f), oblique honeycomb structure (Figure 10g), and element structure (Figure 10h); they verified the advantage of 3D printing in manufacturing ceramic–metamaterial microwave absorbers.

**Figure 11:**
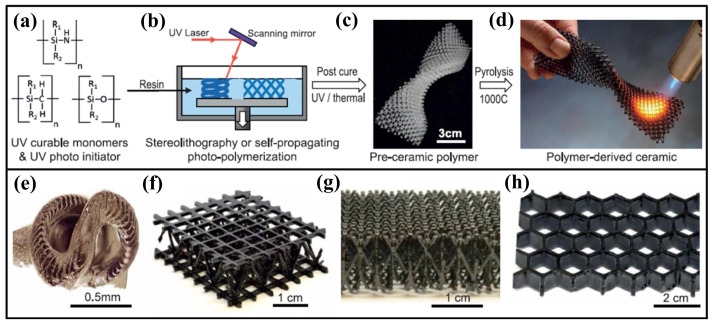
3D printing of polymer-derived ceramics. Reproduced with permission from Ref. [115]. Copyright 2016, Science. (**a**) UV-curable pre-ceramic monomer mixed with photo initiator; (**b**) schematic SLA, wherein the resin is exposed to UV light; (**c**) preparation of pre-ceramic polymer components; (**d**) pyrolysis used to convert polymers into ceramics; (**e**) SLA cork screws; (**f**,**g**) micro crystal lattice created using spreading light polymer waveguide technology; (**h**) honeycomb structure.

The 3D printing technologies currently available for ceramics proceed either by selectively curing photosensitive resins containing ceramic particles, selectively depositing liquid binders onto ceramic particles, or selectively fusing powder beds with lasers. However, these technologies are limited by their slow manufacturing and, in many cases, the time required to remove the additional binder. Based on ceramic powder 3D printing, it is a huge challenge to produce high-quality structural ceramics. Using polymers as precursors for printing ceramics (Figure 11) is a new technology developed only recently. According to the material characteristics of the polymer precursor, different 3D printing technologies are selected for molding, and then the precursor with a specific shape or structure is heated up to a specific temperature in a certain atmosphere to be transformed into a ceramic. Relative to the traditional 3D printing ceramic preparation technology for directly melting ceramic powders, the product obtained by this new technology has higher resolution, a smoother surface, higher density, and allows for easier control of the composition and microstructure [115,116,117,118].

Thus far, many excellent results have been achieved. Miao et al. [119] combined DIW with polymer precursor conversion ceramic technology to obtain SiOC wave-absorbing ceramics. The minimum reflection loss reached −36 dB, and the EAB was 2 GHz. Yao et al. [120] summarized the optimal dielectric constant criterion for non-magnetic materials with a *RL_min_* below −150 dB in theory; they proposed a simple criterion for evaluating microwave absorption properties based on the offset between the dielectric Cole–Cole curve and the theoretical optimal value. A strong and lightweight SiOC ceramic metamaterial with a gyroshell unit structure was prepared by using reduction photopolymerization combined with polymer precursor conversion ceramic technology. It had excellent microwave absorption and mechanical properties; the *RL_min_* was up to −70.3 dB, the EAB crossed the entire X-band, and the specific compressive strength was up to 63.0 MPa/(g/cm^3^) at an ultra-low density of 0.606 g/cm^3^ [120]. This provides a theoretical framework for the structural optimization and actual fabrication of heterogeneous metamaterials.

The precursor molecular structure can be designed, and this natural advantage makes the PDC process more suitable for 3D printing using the photopolymerization mechanism (SLA, DLP). Due to the design of the precursor molecular structure, the introduction of photocurable groups (such as acrylate groups and unsaturated double-bond groups) can provide photocurable properties for the precursor molecule [111].

Zanchetta et al. [121] modified commercially available methicone resin methylsiloxane (MK) through a hydrolysis condensation reaction and obtained a UV-curable poly MK-3-trimethoxysiloxane propyl methacrylate (TMSPM) polysiloxane ceramic precursor, where DPL was used to prepare PDC with a complex structure for the first time, which provided important inspiration for the photocurable 3D printing of various PDCs. Eckel et al. [115] mixed mertan and vinyl (both containing polysiloxane) to obtain ultraviolet (UV)-active pre-ceramic monomer. Based on SLA, they successfully prepared a 3D polymer structure with a complex shape and unit structure and then formed ceramics with uniform shrinkage and almost no pores through pyrolysis. It has been further proven that the photocuring property of ceramic precursors provides a feasible new direction for the preparation of complex structure ceramics by integrating ceramic precursors and 3D printing and opens up new opportunities for the 3D printing of ceramic-based wave-absorbing metamaterials (Figure 11) [115].

**Figure 12:**
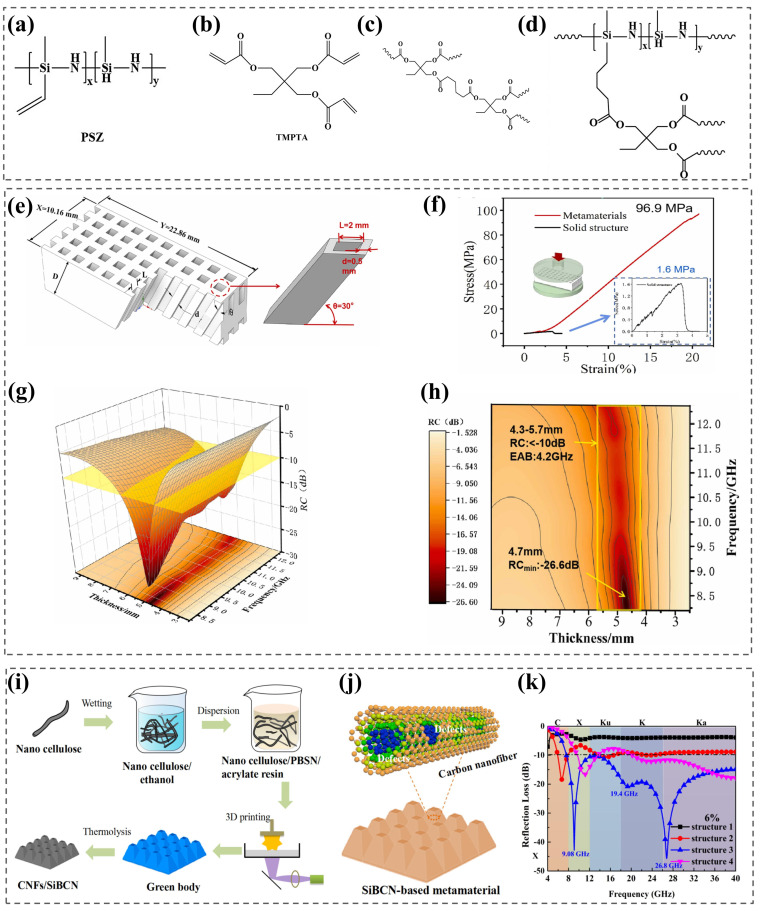
(**a**) Chemical structures of PSZ. Reproduced with permission from Ref. [122]. Copyright 2021, Elsevier. (**b**) Chemical structures of TMPTA. Reproduced with permission from Ref. [122]. Copyright 2021, Elsevier. Chemical structure of the PSZ/TMPTA system after photopolymerization. Reproduced with permission from Ref. [122]. Copyright 2021, Elsevier. (**c**) Photopolymerization between acrylate groups and (**d**) copolymerization between acrylate groups and vinyl groups in PSZ. (**e**) Metamaterial structure design model. Reproduced with permission from Ref. [123]. Copyright 2023, Elsevier. (**f**) Load displacement and compressive strength curves of metamaterials and solid structures. Reproduced with permission from Ref. [123]. Copyright 2023, Elsevier. (**g**,**h**) X-band RC results of direct pyrolysis of metamaterials with different thicknesses at 1500 °C. Reproduced with permission from Ref. [123]. Copyright 2023, Elsevier. (**i**) Schematic diagram of the preparation of CNFs/SiBCN-based materials. Reproduced with permission from Ref. [124]. Copyright 2021, Elsevier. (**j**) Schematic diagram of the CNFs/SiBCN unit structure [124] and (**k**) RL curves of four unit structures containing 6% CNFs after annealing at 1300 °C. Reproduced with permission from Ref. [124]. Copyright 2021, Elsevier.

Pan et al. [122] mixed a polysilazane (PSZ) (Figure 12a) ceramic precursor with trimethylpropane triacrylate (TMPTA) (Figure 12b) to obtain raw materials for SLA (Figure 12d) and prepared a SiCN ceramic metamaterial absorber with a honeycomb structure and an EAB of 3.02 GHz in the X-band. The *RL_min_* was up to −49 dB. In addition, the prepared ceramics also had high density and excellent mechanical properties.

Wang et al. [123] added poly-borosilazane into the premixed resin solution, used ultrasound to completely dissolve the precursor, and finally removed the bubbles inside the material to obtain a poly-borosilane photosensitive resin material as a ceramic precursor that could be used for DPL. By combining DPL with polymer precursor conversion ceramic technology, SiBCN metamaterials with excellent wave-absorbing properties were prepared (Figure 12e). X-band effective absorption was achieved, and the *RL_min_* reached −26.6 dB when the metamaterial was 4.3~5.7 mm in thickness (Figure 12g,h). In addition, the metamaterial had a porosity of 64.25% and a specific compressive strength of 96.9 MPa/(g/m^3^), which combined the merits of light weight and high mechanical strength (Figure 12f) [123]. This method of fabricating an EMW-absorbing metamaterial structure achieves the integration of structure and function and simply and effectively meets the requirements of EMW-absorbing devices, which should be light and thin and have a wide bandwidth and strong absorption performance.

Liu et al. [124] prepared polyborosilazane ceramic precursor that could be used for DPL by mixing polyborosilazane with photosensitive resin and mixed carboxyl-functionalized nanocellulose (NC-COOH) into the printing liquid. During the process of pyrolysis and the conversion of printing blanks into ceramics, defection-rich CNFs were generated in situ. Based on the 3D-printed metamaterial structure design and the microstructure design of the polymer precursor, a CNFs/SiBCN metamaterial (Figure 12i,j) with excellent wave absorption performance was prepared, which achieved effective absorption in an ultra-wide band of 7.6~40 GHz with an *RL_min_* as low as −46 dB (Figure 12k) [124]. This sheds new light on the development of ceramic-based wideband electromagnetic wave-absorbing materials.

### 4.3. High-Temperature Wave-Absorbing Ceramic Metamaterials Based on 3D Printing

Most of the existing 3D-printed ceramic-based wave-absorbing metamaterials are not high-temperature-resistant. It is of great importance that we develop temperature-insensitive ceramic-based wave-absorbing metamaterials.

**Figure 13:**
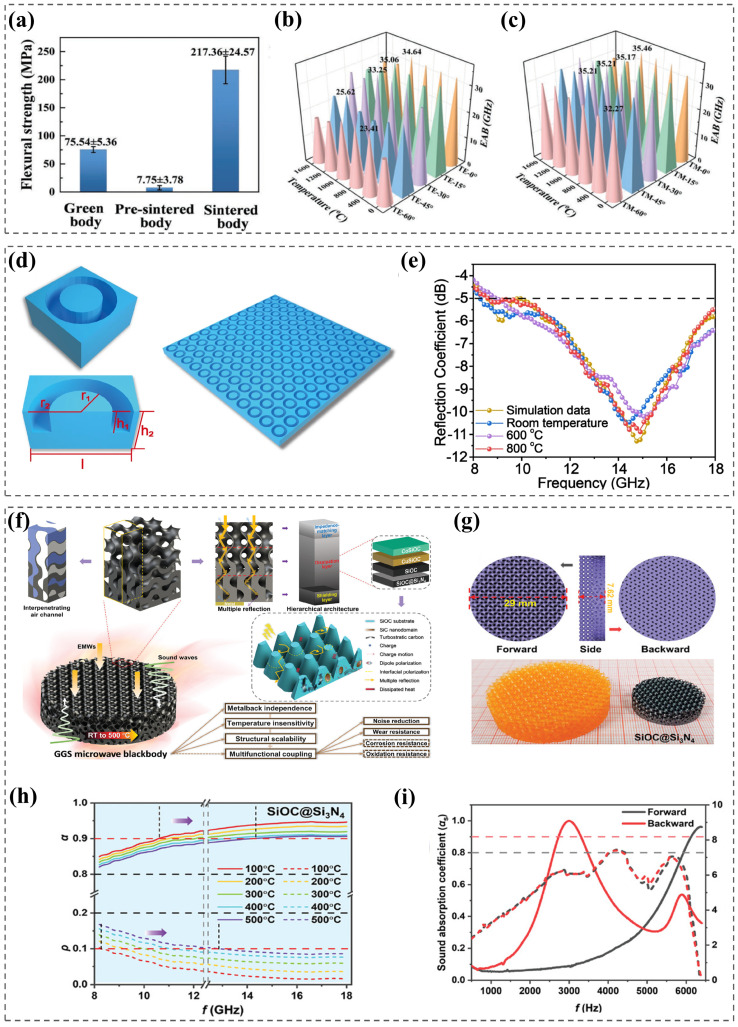
(**a**) Flexural strength of SiC sintered body. Reproduced with permission from Ref. [41]. Copyright 2022, Wiley. (**b**) EAB under transverse electric polarization. Reproduced with permission from Ref. [41]. Copyright 2022, Wiley. (**c**) EAB under transverse magnetic polarization. Reproduced with permission from Ref. [41]. Copyright 2022, Wiley. (**d**) SiOC ceramic metamaterial unit and array structure model. Reproduced with permission from Ref. [42]. Copyright 2022, Springer Nature. (**e**) Comparison between measured and simulated RC data of SiOC ceramic metamaterials at different temperatures. Reproduced with permission from Ref. [42]. Copyright 2022, Springer Nature. (**f**) Schematic diagram of metamaterial absorption mechanism of GGS structure. Reproduced with permission from Ref. [43]. Copyright 2022, Wiley. (**g**) EMW absorption capacity in the range of 373~773 K. Reproduced with permission from Ref. [43]. Copyright 2022, Wiley. (**h**) Front, side and rear views of the acoustic test structure configuration (top) and corresponding green body and SiOC@Si_3_N_4_ specimen (bottom). Reproduced with permission from Ref. [43]. Copyright 2022, Wiley. (**i**) Experimental sound absorption index and sound attenuation index. Reproduced with permission from Ref. [43]. Copyright 2022, Wiley.

Zhou et al. [41] used SLA to prepare a SiC ceramic metamaterial, which had both good mechanical strength and excellent wave absorption performance. Densification of SiC ceramics was achieved by this preparation method, so that they could withstand 217.36 MPa loads (Figure 13a). In the wide temperature range from room temperature to 1873 K, excellent broadband absorption performance was maintained, and an EAB of 6.96~40.00 GHz was achieved at room temperature; the EAB was 33.04 GHz. At 1873 K, the EAB was maintained at 28.78 GHz (10.89~39.67 GHz). In addition, from room temperature to 1873 K, the material was also insensitive to polarization and differing incidence angle and achieved wideband microwave absorption from 0~60° oblique incidence (Figure 13b,c) [41]. This study provides a general strategy for realizing ultra-wideband and high-temperature EMW absorption through the 3D printing of SiC ceramic metamaterial structures with stereo-lithography, which has great application potential in the aerospace field.

By introducing acrylic groups and silicone vinyl groups, Zhou et al. [42] obtained precursors of polysiloxane ceramics with high yield and decent photocuring performance (Figure 13d). Combined with DPL, cross-helical-array SiOC ceramic-based wave-absorbing metamaterials were prepared, which achieved a low reflection coefficient and broadband absorption from room temperature to 1073 K. At 1073 K, the *RL_min_* was −10.89 dB, and the EAB below −5 dB was 9.35 GHz (8.65~18.00 GHz) (Figure 13e). In addition, the effective absorption frequency band was extended by adjusting the cell structure design [42]. This study combines PDC with 3D printing technology to provide a new method for manufacturing tunable EMW-absorbing ceramic materials and wide-absorption-band metamaterials with high design flexibility and high efficiency; it provides a novel and effective method for the design and manufacture of EMW-absorbing ceramic metamaterials.

Yao et al. [43] combined the structural design of gradient gyro sheath units (GGSs) (Figure 13f) with the 3D printing of polymer-derived SiOC (PDC-SiOC) ceramics coated with Si_3_N_4_ and prepared a wideband-absorbent GGS structure within SiOC@Si_3_N_4_ wave-absorbing ceramic metamaterials. The microwave absorption intensity averaged up to 91.3% across the entire X-Ku band, including high frequencies above 18 GHz, and was temperature-insensitive from room temperature to 773 K (Figure 13g). In addition, the SiOC@Si_3_N_4_ metamaterial with a GGS structure also displayed a noise reduction effect (Figure 13h,i) and ultra-high wear resistance, realizing multi-functional coupled broadband microwave absorption [43].

## 5. Conclusions and Prospects

High-temperature-resistant electromagnetic wave absorbers based on ceramic materials have been analyzed herein, mainly through their material composition and structure. In addition, the applications of metamaterials and 3D printing technologies in this area have been introduced. They are summarized as follows.

Regarding material composition, the wave absorption performance of single ceramic materials is not up to requirements, which becomes more obvious at high temperatures. Therefore, composite ceramic materials are often used to improve their wave-absorbing properties. High-temperature wave-absorbing composite ceramic materials are usually composed of a matrix of low-dielectric wave-transmitting ceramics doped with magnetic loss-type or conductance loss-type materials. Impedance matching is optimized under the synergistic effect of various losses, and the interface polarization loss is increased through the rich interface between different materials, which both improve wave-absorbing performance.

In terms of structural characteristics, the absorption performance of the material can be improved through structural design. A porous structure can enhance the repeated absorption and scattering loss of electromagnetic waves inside the absorbing material. A core–shell structure can increase the interface and hierarchy of materials. The multilayer structure provides a rich interface and increases the interface polarization loss, and the reasonable adjustment of the electromagnetic parameters of the matching layer and the loss layer can produce a wider absorbing bandwidth. The dielectric constant and permeability of metamaterials can be adjusted within a wide range, which can greatly expand the absorption bandwidth.

Three-dimensional printing, as an advanced manufacturing technology with high flexibility, enables the rapid manufacturing of metamaterials with 3D complex structures. In the field of electromagnetic wave absorption, the use of 3D printing technology to prepare ceramic-based wave-absorbing metamaterials has made remarkable progress, but there are still some limitations. Metamaterials usually require multiple materials with which to build complex structures and achieve electromagnetic properties, which can slow down the 3D printing process. In addition, existing 3D printing technologies face difficulties in meeting the requirements of high precision and suitability for large-scale manufacturing.

Polymer precursor conversion ceramic technology is one of the key methods with which ceramic matrix composites can be prepared. Through the structural design and fine synthesis of precursor material molecules and a controlled pyrolysis process, ceramic materials with an adjustable element composition and a crystalline phase domain structure can be obtained. A variety of molding methods can endow polymer-precursor converted ceramics with a varied morphology. In addition, the soluble or meltable nature of the ceramic precursor is suitable for combination with 3D printing technology on the one hand; on the other hand, it is conducive to the uniform distribution of the external absorbers in the precursor in order to achieve ceramic absorbing materials with synergistically enhanced loss performance.

Once the absorber is formed, its electromagnetic parameters, such as absorption bandwidth and minimum reflection loss, are basically fixed; thus, it is difficult for it to meet changing needs in a dynamic environment. Combining metamaterials with materials with adjustable properties (anisotropic liquid crystals, graphene, phase-change VO_2_, etc.) in order to build new types of high-temperature-resistant electromagnetic wave-absorbing materials is an emerging research direction, which will play a huge role in the field of intelligent stealth.

## Figures and Tables

**Table 1:** High-temperature absorption properties of carbon/ceramic composites (in the X-band).

Samples	Temperature Range (K)	Thickness (mm)	*RL*_min_ (dB)	EAB (GHz)	References
RGO/Si_3_N_4_	323~873	4.3	−14.0 at 873 K	4.2 at 873 K	[63]
RGO/SiOC	293~673	2.3	−50.1 at 673 K	3.9 at 673 K	[32]
MWCNTs/SiO_2_	373~773	3.5	−39.4 at 773 K	4.2 at 773 K	[64]
SiCnw/C/Si_3_N_4_	298~873	2.9	−39.7 at 873 K	4.2 at 873 K	[65]
SiCf/BN/AlPO_4_/MWCNT	298~873	3.1	−22.0 at 873 K	3.7 at 873 K	[66]
CF–SiCnw/Si_3_N_4_	298~1073	2.0	−20.4 at 1037 K	4.2 at 1037 K	[67]
RGO–SiCnw/SiBCN	298~873	3.6	−39.1 at 873 K	4.2 at 873 K	[68]
CNT/SiC	298~873	1.8	−51.0 at 873 K	2.0 at 873 K	[34]

**Table 2:** High-temperature absorption properties of metal oxide/ceramic composites (in the X-band).

Samples	Temperature Range (K)	Thickness (mm)	*RL_min_* (dB)	EAB (GHz)	References
Al doped ZnO-ZrSiO_4_	298~773	2.86	~−15.0 at 773 K	3.4 at 773 K	[36]
HfO_2_-SiBCN	298~1073	1.58	−44.8 at 1073 K	2.4 at 1073 K	[37]
Al_2_O_3_-MoSi_2_-Cu	298~773	1.70	−18.0 at 773 K	2.4 at 773 K	[83]
NiO-SiC	373~773	/	~−20.0 at 773 K	4.2 at 773 K	[84]
Fe_3_O_4_@ZrO_2_	298~773	/	~25.0 at 773 K	/	[35]
graphene@Fe_3_O_4_/SiBCN	298~873	2.35	−46.0 at 873 K	3.9 at 873 K	[20]
ZnO-MWCNT/SiO_2_	323~673	2.50	−13.0 at 673 K	3.4 at 673 K	[85]

**Table 3:** High-temperature wave absorption properties of ceramic materials (in the X-band).

Samples	Temperature Range (K)	Thickness (mm)	*RL_min_* (dB)	EAB (GHz)	References
Fe-doped SiBCN	298~1158	2.0	−12.6 at 1158 K	3.2 at 1158 K	[39]
TiN/BN	298~873	3.0	−16.7 at 873 K	2.7 at 873 K	[87]
Si_3_N_4_–SiC/SiO_2_	298~873	3.3	−35.9 at 873 K	4.0 at 873 K	[88]
Si_3_N_4_/SiC	473~1273	2.8	≤−15.0 at 1273 K	4.2 at 1273 K	[38]
SiCnw/SiCw	298~873	<5.0	−15.0 at 873 K	3.0 at 873 K	[89]
MOF/SiBCN	298~873	2.6	−30.3 at 873 K	4.0 at 873 K	[40]
Zn Al_2_O_3_	298~873	2.5	−12.1 at 873 K	3.0 at 873 K	[90]
Ni-doped SiC	373~673	2.1	−48.1 at 673 K	4.2 at 673 K	[91]
Co-doped SiC	323~573	1.7	−44.7 at 573 K	3.0 at 573 K	[92]

**Table 4:** Wave-absorbing properties of traditional ceramic-based wave-absorbing metamaterials at high temperatures.

Samples	Temperature Range (K)	Thickness (mm)	Absorbing Bandwidth	Unit Cell	References
TiB_2_/Al_2_O_3_/TiB_2_	273~1073	2.7	10.6~11.0 GHz (*RL* < −10 dB)	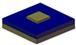	[95]
La_0.5_Sr_0.5_CoO_3_/Al_2_O_3_	373~773	1.7, 1.8	8.2~12.4 GHz (*RL* < −10 dB)	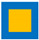 , 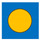	[96]
MoSi_2_/Al/water glass	298~873	3.0	7.0~13.0 GHz (*RL* < −10 dB)	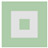	[81]
Al_2_O_3_	273~1073	1.5	10.0~18.0 GHz (*RL* < −5 dB)	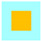	[97]
TiB_2_/Al_2_O_3_/MgAl_2_O_4_	298~1373	6.0	8.2~18.0 GHz (*RL* < −5 dB)	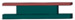	[98]
SiC_f_/Si_3_N_4_	298~773	5.0	8.0~18.0 GHz (*RL* < −5 dB)	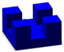	[99]
